# Supplementary material for: Gait Variability to Phenotype Common Orthopedic Gait Impairments Using Wearable Sensors
Source: Sensors (Basel). 2022 Nov 29;22(23):9301. doi: 10.3390/s22239301 (PMC9739785; doi:10.3390/s22239301)
Supplement: Supplementary file 1 [file sensors-22-09301-s001.zip › sensors-2045878-supplementary.pdf]

Supplementary file S1. Participants' demographic characteristics.

(HC; Healthy Control, LSS; Lumbar Spinal Stenosis, KOA; Knee Osteoarthritis, BMI; Body Mass Index, SF-36; 36-item short-form).

	HC	LSS	KOA
n	10	10	10
Female	5	7	6
Age (yrs)	61.2 (9.9)	70.3 (9.4)	63.9 (8.1)
BMI (kg/m2)	27.2 (4.1)	29.6 (4.1)	33.2 (8.4)
SF-36 Physical Function	88.5 (10.6)	40.5 (25.1)	32.5 (14.4)

Supplementary file S2. Changes in parameters from minute-to-minute analysis of 6MWT. (HC; Healthy Control, LSS; Lumbar Spinal Stenosis, KOA; Knee Osteoarthritis, CV; Coefficient of Variation).

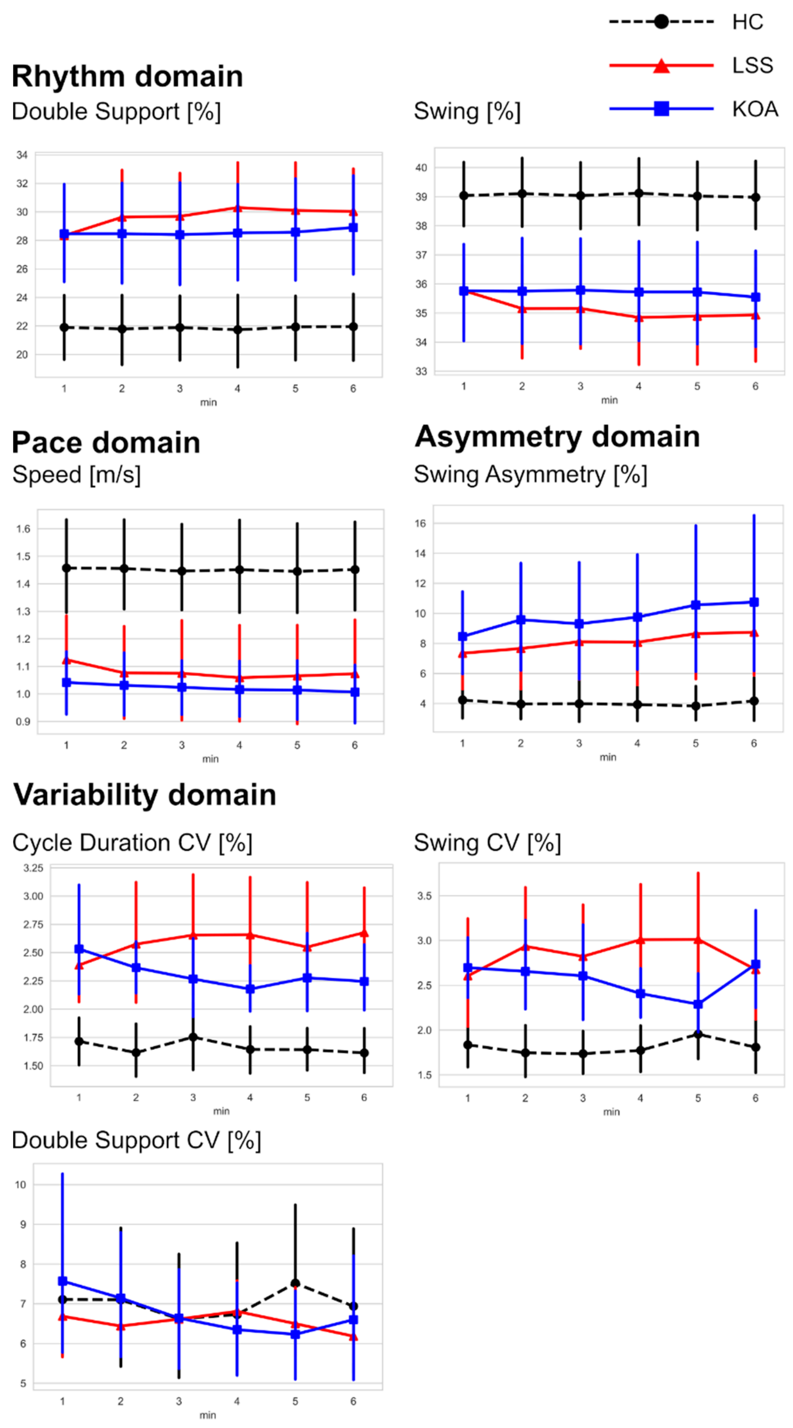

Supplementary file S3. Radar plot illustrating the gait parameters extracted from analysis of the entire 6MWT (Data comparison between limbs). The central black dots with connecting dashed lines represents HC data. This is compared to LSS (blue squares and lines) and KOA (red triangles and lines) with deviation along the axis radiating from the center of the plot representing the standard deviations (range; from -3 SD to +6SD) from HC. (HC; Healthy Control, LSS; Lumbar Spinal Stenosis, KOA; Knee Osteoarthritis, CV; Coefficient of Variation, SD; Standard Deviations).

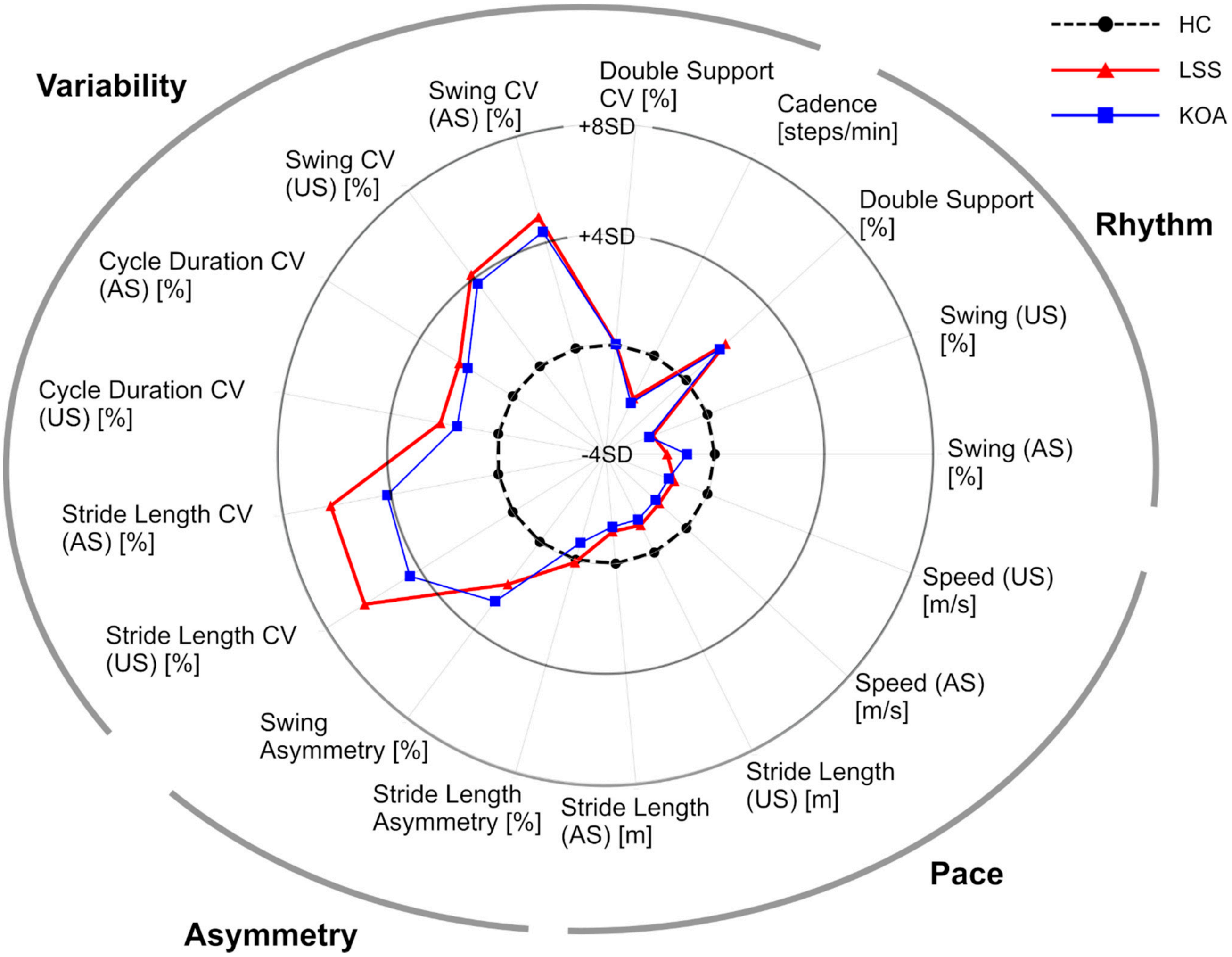

Supplementary file S4. The gait parameters extracted from analysis of the entire 6MWT (Data comparison between limbs). Significant p values ($p < 0.05$) are shown in bold. (HC; Healthy Control, LSS; Lumbar Spinal Stenosis, KOA; Knee Osteoarthritis, CV; Coefficient of Variation, US; Unaffected side limb, AS; Affected side limb).

					p value		
Domain	parameter	HC	LSS	KOA	HC-LSS	HC-KOA	LSS-KOA
Rhythm	Swing (US) [%]	39.1 (2.0)	34.7 (2.9)	34.4 (2.2)	**0.001**	**0.001**	0.900
	Swing (AS) [%]	39.1 (2.0)	35.5 (2.8)	37.0 (4.4)	0.052	0.341	0.564
Pace	Speed (US) [m/s]	1.5 (0.3)	1.1 (0.3)	1.0 (0.2)	**0.012**	**0.003**	0.857
	Speed (AS) [m/s]	1.5 (0.3)	1.1 (0.3)	1.0 (0.2)	**0.008**	**0.003**	0.900
	Stride Length (US) [m]	1.4 (0.2)	1.2 (0.3)	1.2 (0.2)	0.068	**0.024**	0.875
	Stride Length (AS) [m]	1.4 (0.2)	1.2 (0.3)	1.2 (0.2)	**0.048**	**0.021**	0.900
Variability	Stride Length CV (US) [%]	3.6 (0.3)	5.3 (0.7)	4.8 (0.8)	**0.001**	**0.001**	0.174
	Stride Length CV (AS) [%]	3.6 (0.3)	5.3 (0.8)	4.7 (0.8)	**0.001**	**0.003**	0.163
	Cycle Duration CV (US) [%]	1.9 (0.7)	3.4 (0.9)	2.9 (0.8)	**0.001**	**0.023**	0.499
	Cycle Duration CV (AS) [%]	1.9 (0.7)	3.4 (1.0)	3.2 (0.9)	**0.002**	**0.008**	0.810
	Swing CV (US) [%]	1.9 (0.5)	3.8 (1.3)	3.6 (1.0)	**0.001**	**0.002**	0.900
	Swing CV (AS) [%]	1.9 (0.5)	4.2 (1.7)	3.9 (1.2)	**0.001**	**0.002**	0.875

Supplementary file S5. Changes in parameters from minute-by-minute analysis of 6MWT (Data comparison between limbs). The * identifies significant difference between LSS and KOA ($p < 0.05$). (HC; Healthy Control, LSS; Lumbar Spinal Stenosis, KOA; Knee Osteoarthritis, CV; Coefficient of Variation).

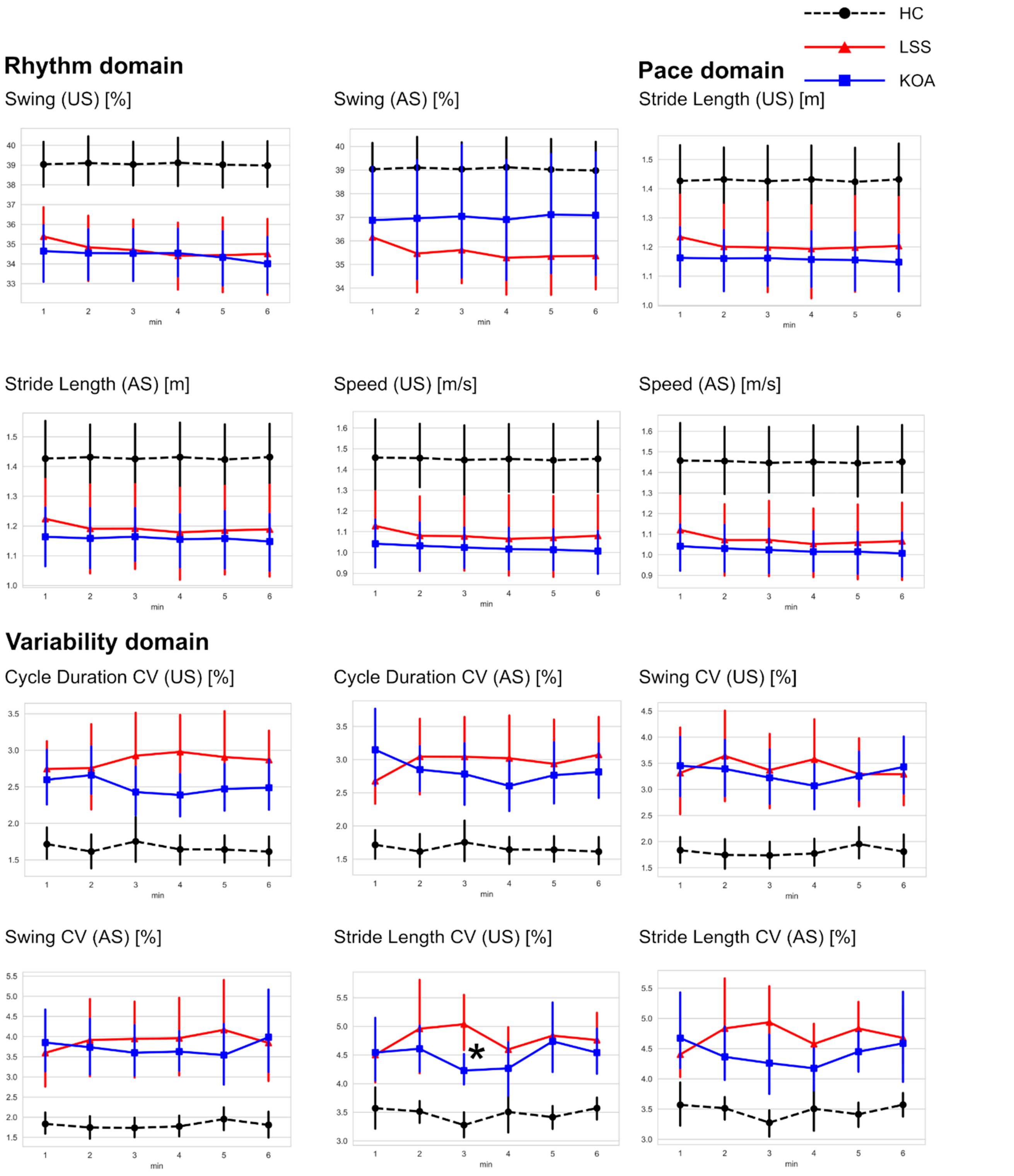

Supplementary file S6. Details on gait parameter extraction.

The Shimmer3 wearable sensor platform (Shimmer Sensing, Dublin, Ireland) was the IMU used for data collection. An IMU sensor was placed on the dorsal surface of the participant's right and left foot using shimmer straps. Each IMU sensor consists of a 3D accelerometer, a 3D gyroscope, a 3D magnetometer. Data were sampled at 102.4 Hz and hardware synced by the control software. We used validated algorithms (a rule-based stance phase event detection algorithm) [B. Mariani, et al. Gait Posture. 37 (2013) 229–234., B. Mariani, et al. J. Biomech. 43 (2010) 2999–3006.] to extract the spatiotemporal gait parameters from the IMU sensors. Prior to processing, data were resampled to 200Hz using linear interpolation to be consistent with the validated algorithms [W. Zhang, et al. Sensors. 18 (2018) 3322.]. Gait cycles were detected based on the timing of two consecutive foot-flats [B. Mariani, et al. Gait Posture. 37 (2013) 229–234.]. Velocity and position of the foot were extracted by the numerical integration of the gravity-corrected acceleration data and drift corrected using the ZUPT method [E. Foxlin, et al. IEEE Comput. Graph. Appl. (2005) 38–46.]. Heel strike and lift off angles were estimated based on the de-drifted angular velocity data [B. Mariani, et al. IEEE Trans. Biomed. Eng. 59 (2012) 3162–3168.]. Maximum angular velocity of the foot and various temporal parameters were extracted from the angular velocity signals [B. Mariani, et al. Gait Posture. 37 (2013) 229–234.]. Cycles with a turning angle between two foot-flats less than 20 degrees were considered as straight walking cycles [B. Mariani, et al. J. Biomech. 43 (2010) 2999–3006.].
